# Management of Inflammatory Bowel Disease in Pregnancy: A Practical Approach to New Guidelines

**DOI:** 10.1155/2016/9513742

**Published:** 2016-07-11

**Authors:** V. Huang, Y. Leung, G. C. Nguyen, C. H. Seow

**Affiliations:** ^1^Department of Medicine, University of Alberta, Edmonton, AB, Canada T6G 2X8; ^2^Department of Medicine, University of Calgary, Calgary, AB, Canada T2N 4Z6; ^3^Mount Sinai Hospital Centre for Inflammatory Bowel Disease, University of Toronto, Toronto, ON, Canada M5G 1X5; ^4^Department of Community Health Sciences, University of Calgary, Calgary, AB, Canada T2N 4Z6

Inflammatory bowel disease (IBD) affects patients in their adolescent and adult years of life, during a time when they may be contemplating pregnancy or are pregnant. Maternal IBD and, specifically, active IBD during pregnancy are associated with an increased risk of adverse maternal and fetal outcomes including prematurity, low birth weight, and cesarean delivery [1]. Thus optimization and management of IBD before and during pregnancy are important in improving maternal and fetal outcomes. Nevertheless, patients and physicians are challenged on balancing the benefits of therapy for active maternal IBD with the potential risks of these therapies to the fetus.

The medical literature has demonstrated that significant gaps in knowledge about the management of IBD during pregnancy exist in both patients and their physicians, resulting in misperceptions about IBD therapies during pregnancy [2–4]; “voluntary childlessness” as a consequence of fear of infertility, the ability to cope with the pregnancy in the presence of disease, heritability of the disease, and adverse fetal outcomes [2–4]; medication nonadherence [3–7]; and misdirected physician-led modification or cessation of therapies in this cohort [7–9]. Further, in a recent Canadian survey of physicians, less than 50% of surveyed physicians felt comfortable managing IBD during pregnancy [10]. In Canada, family physicians and nurses are often the primary initial contact for the IBD patient in their journey from preconception to pregnancy. Once pregnant, women in the Canadian healthcare system are cared for by a multidisciplinary team that includes family physicians, obstetricians, nurses, and midwives. Acknowledging the knowledge gap and changing landscape of IBD management, “The Toronto Consensus Statements for the Management of Inflammatory Bowel Disease in Pregnancy” were developed, integrating up-to-date evidence vetted by the GRADE approach and tailored to North American clinical practice [11].

“The Toronto Consensus Statements for the Management of Inflammatory Bowel Disease in Pregnancy” [11] consist of 29 recommendations (27 statements, of which two statements have two parts, i.e., 4A, 4B, 10A, and 10B) spanning preconception, pregnancy, and postpartum care. The recommendations can be viewed in Table 1 or within the hyperlink provided: https://www.cag-acg.org/images/publications/PIIS0016508515017734.pdf. The working group comprised experts from multiple disciplines which included (1) gastroenterologists with expertise in the management of IBD; (2) obstetricians; (3) specialists in maternal-fetal medicine; (4) pharmacologists; and (5) female IBD patient representation. The consensus panel was also well represented by Motherisk, an internationally recognized program that provides information and advice to pregnant women and their healthcare providers on the safety of medications during pregnancy and breastfeeding. As adverse events such as preterm delivery can lead to higher rates of infant mortality, appropriate and timely diagnostic and treatment interventions during pregnancy can be considered lifesaving measures. Taking this into consideration and the highly favorable benefit-harm profile, the working group designated 21 of the 29 recommendations as strong despite the very low quality of evidence presented.

In contrast to previously published guidelines, the Toronto consensus is very explicit in its recommendations for management of flares during pregnancy. These recommendations are stratified by disease phenotype and current medication(s) at the time of the flare. Statements 14–16 emphasize the use of induction agents that include corticosteroid therapy or anti-TNF therapy. Additionally, the consensus statements help to guide care providers on the use of concomitant thiopurine therapy, making the distinction between a patient who is naïve to thiopurine therapy in the event of a flare (Statement 16) and the scenario of a patient already on combination therapy (Statement 11).

Recognizing that controversy surrounds the use of anti-TNF therapy, the Toronto consensus provides clear recommendations that anti-TNF therapy is to be continued throughout pregnancy (Statement 10A). This differs from various editorials, guidelines, and review articles that have focused on discontinuation of anti-TNF in the late second trimester or early third trimester [12–14]. There is no doubt that the current Toronto guidelines place disease remission as top priority based on solid evidence that inactive disease portends the best prognosis for both maternal and fetal health. Further, consensus members discussed that the common practice of modifying the dosing schedule during the third trimester (e.g., providing an infusion of infliximab at weeks 30–32 and adalimumab at weeks 34–36 and then resuming therapy postpartum) to minimize drug hiatus may help minimize disease flare in the mother but does not minimize fetal exposure, as transplacental drug transfer is very efficient in the second and third trimesters. There is, however, little evidence of an increased risk of infection or developmental delay (at least in the short-term) with continued anti-TNF therapy. However, recognizing a variety of individual factors such as theoretical concerns or strong patient preference, Statement 10B makes the conditional recommendation that, in order to minimize fetal exposure, one could administer the last dose at 22 to 24 weeks of gestation in patients who are at low risk for relapse. Strict criteria for determining patients who are at low risk for relapse are explicitly stated and include sustained symptomatic remission during the 12 months before conception, no active disease on endoscopy or imaging during the preconception period, no prior secondary loss of response to anti-TNF therapy or dose escalation, demonstrated therapeutic levels of anti-TNF therapy, no prior intestinal resections, and no hospitalizations in the past 36 months [15].

These statements make strong recommendations for objective assessments before conception and close monitoring during pregnancy, though it is recognized that some jurisdictions may have limited access to endoscopy, specialized gastroenterology care, or high-risk obstetrical consultation. However, these guidelines provide a framework from which healthcare providers can make decisions regarding the management of IBD during pregnancy and provide a common algorithm that can be followed by the multidisciplinary care team that manages the pregnant woman with IBD.

*V. Huang* *Y. Leung* *G. C. Nguyen* *C. H. Seow*

## Figures and Tables

**Table 1:** Summary of consensus recommendations for the management of IBD in pregnancy.

*Impact of IBD during pregnancy: role of disease management*	
*Statement #1*: We recommend that women with IBD of reproductive age receive preconception counseling to improve pregnancy outcomes. *GRADE: Strong recommendation, very low-quality evidence. *	
*Statement #2*: In women with IBD who are contemplating pregnancy, we recommend objective disease evaluation prior to conception in order to optimize disease management. *GRADE: Strong recommendation, very low-quality evidence. *	
*Statement #3*: In women with ulcerative colitis who are contemplating pregnancy and taking a 5-ASA formulation containing di-butyl phthalate (DBP), we suggest switching to a 5-ASA drug without DBP. *GRADE: Conditional recommendation, very low-quality evidence. *	
*Statement #4A*: In women with IBD who are taking methotrexate and contemplating pregnancy, we recommend stopping methotrexate at least 3 months prior to attempting to conceive to minimize the risk of teratogenicity. *GRADE: Strong recommendation, very low-quality evidence. *	
*Statement #4B*: If a woman becomes pregnant while taking methotrexate, we recommend immediate discontinuation of methotrexate and referral for obstetric counseling. *GRADE: Strong recommendation, very low-quality evidence. *	
*Statement #5*: In pregnant women with active or complicated IBD, we recommend consultation with an obstetrician, preferably one affiliated with a high-risk obstetrics program. *GRADE: Strong recommendation, very low-quality evidence. *	
*Statement #6*: In pregnant women with IBD, we recommend their IBD be managed by a gastroenterologist throughout pregnancy. *GRADE: Strong recommendation, very low-quality evidence. *	
*Statement #7*: In pregnant women who require hospitalization for their IBD, we recommend transfer to a tertiary center with access to a gastroenterologist and an obstetrician, preferably one affiliated with a high-risk obstetrics program. *GRADE: Strong recommendation, very low-quality evidence. *	
*Medical management of IBD during pregnancy*	
*Statement #8*: In pregnant women with IBD on oral and/or rectal 5-ASA maintenance therapy, we recommend continuation of 5-ASA therapy throughout pregnancy. *GRADE: Strong recommendation, very low-quality evidence. *	
*Statement #9*: In pregnant women with IBD on thiopurine maintenance therapy, we recommend continuation of thiopurine therapy throughout pregnancy. *GRADE: Strong recommendation, very low-quality evidence. *	
*Statement #10A*: In pregnant women with IBD on anti-tumour necrosis factor (anti-TNF) maintenance therapy, WE RECOMMEND CONTINUATION of anti-TNF therapy. *GRADE: Strong recommendation, very low-quality evidence. *	
*Statement #10B*: In select pregnant women at low-risk for an IBD relapse who have a compelling reason to discontinue anti-TNF therapy TO MINIMIZE FETAL EXPOSURE, we suggest administering the last dose at 22–24 weeks gestation. *GRADE: Conditional recommendation, very low-quality evidence.*	
*Statement #11*: In pregnant women with IBD on combination anti-TNF and thiopurine therapy, we suggest that the decision to switch to monotherapy should be individualized. *GRADE: Conditional recommendation, very low-quality evidence. *	
*Statement #12*: In pregnant women with ulcerative colitis who have a mild-to-moderate disease flare while on 5-ASA maintenance therapy, we recommend combination 5-ASA oral and rectal therapy be optimized to induce symptomatic remission. *GRADE: Strong recommendation, very low-quality evidence. *	
*Statement #13*: In pregnant women with Crohn's disease with perianal sepsis requiring antibiotic therapy, we suggest metronidazole and/or ciprofloxacin. *GRADE: Conditional recommendation, very low-quality evidence. *	
*Statement #14*: In pregnant women with IBD who have a disease flare on optimal 5-ASA or thiopurine maintenance therapy, we recommend treatment with systemic corticosteroids or anti-TNF therapy to induce symptomatic remission. *GRADE: Strong recommendation, very low-quality evidence. *	
*Statement #15*: In pregnant women with IBD who have a corticosteroid-resistant flare, we recommend starting anti-TNF therapy to induce symptomatic remission. *GRADE: Strong recommendation, very low-quality evidence. *	
*Statement #16*: In pregnant women with IBD who are thiopurine naïve and starting anti-TNF therapy, we suggest anti-TNF monotherapy over combination therapy with anti-TNF and thiopurine therapy. *GRADE: Conditional recommendation, very low-quality evidence. *	
*Statement #17*: In pregnant women hospitalized for IBD, we recommend anticoagulant thromboprophylaxis during hospitalization over no prophylaxis. *GRADE: Strong recommendation, very low-quality evidence. *	
*Imaging, endoscopy and surgery for IBD during pregnancy*	
*Statement #18*: In pregnant women with suspected IBD or IBD flare, we recommend use of flexible sigmoidoscopy or colonoscopy if the results will impact the antenatal management of the IBD. *GRADE: Strong recommendation, very low-quality evidence. *	
*Statement #19*: In pregnant women with suspected IBD or IBD flare, we recommend limiting radiologic investigations to the use of sonography and magnetic resonance imaging where possible. *GRADE: Strong recommendation, very low-quality evidence. *	
*Statement #20*: In pregnant women with IBD, we recommend that urgent surgery to manage IBD complications not be delayed solely due to pregnancy. *GRADE: Strong recommendation, very low-quality evidence. *	
*Issues around delivery for pregnant women with IBD*	
*Statement #21*: For pregnant women with IBD, we recommend basing the decision for cesarean delivery on obstetrical considerations and not IBD diagnosis alone. *GRADE: Strong recommendation, very low-quality evidence. *	
*Statement #22*: For pregnant women with IBD who have undergone an ileal pouch anal anastomosis (IPAA) procedure, we suggest consideration of cesarean delivery to reduce the risk of anal sphincter injury, in consultation with an obstetrician and surgeon. *GRADE: Conditional recommendation, very low-quality evidence. *	
*Statement #23*: For pregnant women with Crohn's disease who have active perianal disease, we recommend cesarean delivery over vaginal delivery to reduce the risk of perianal injury. *GRADE: Strong recommendation, very low-quality evidence. *	
*Statement #24*: For pregnant women with IBD who have undergone cesarean delivery, we recommend anticoagulant thromboprophylaxis during hospitalization over no prophylaxis. *GRADE: Strong recommendation, very low-quality evidence. *	
*Breastfeeding and vaccination of newborns of women with IBD*	
*Statement #25*: In women with IBD, we suggest that use of 5-ASA, systemic corticosteroids, thiopurines, or anti-TNF therapy should not influence the decision to breastfeed, and breastfeeding should not influence the decision to use these medications. *GRADE: Conditional recommendation, very low-quality evidence. *	
*Statement #26*: In women with IBD who are breastfeeding, we suggest avoiding methotrexate therapy. *GRADE: Conditional recommendation, very low-quality evidence. *	
*Statement #27*: For newborns of women who were on anti-TNF therapy during pregnancy, we recommend against administration of live vaccinations within the first 6 months of life. *GRADE: Strong recommendation, very low-quality evidence.*	
